# Alcohol Consumption Assessed by a Biomarker and Self-Reported Drinking in a Sample of Pregnant Women in the South of Europe: A Comparative Study

**DOI:** 10.3390/toxics11110930

**Published:** 2023-11-16

**Authors:** Isabel Corrales-Gutierrez, Diego Gomez-Baya, Fatima Leon-Larios, Rocío Medero-Canela, Emilia Marchei, Ramon Mendoza-Berjano, Óscar García-Algar

**Affiliations:** 1 Foetal Medicine Unit, University Hospital Virgen Macarena, 41009 Seville, Spain; icorrales@us.es; 2 Department of Surgery, Faculty of Medicine, University of Seville, 41009 Seville, Spain; 3 Department of Social, Developmental and Educational Psychology, University of Huelva, 21007 Huelva, Spain; ramon@dpsi.uhu.es; 4 Research Group on Health Promotion and Development of Lifestyle across the Lifespan, University of Huelva, 21007 Huelva, Spain; 5 Nursing Department, Faculty of Nursing, Physiotherapy and Podiatry, University of Seville, 41009 Seville, Spain; 6 Multiprofessional Teaching Unit for Family and Community Care in Huelva, Huelva Costa Condado Campiña Health District, 21001 Huelva, Spain; rociomek@hotmail.com; 7 Instituto Superiore di Sanitá, 00161 Roma, Italy; emilia.marchei@iss.it; 8 Neonatology Unit, Hospital Clinic, Universitat de Barcelona, 08028 Barcelona, Spain; ogarciaa@clinic.cat

**Keywords:** fetal alcohol spectrum disorders, pregnancy, alcohol consumption, Spain, biomarker

## Abstract

(1) Background: Alcohol consumption during pregnancy is a major concern, particularly in Europe and North America. Its prevalence has so far been under-researched. In most studies, the determination of this consumption may be underestimated, as it is based on the information obtained from questionnaires rather than from biomarkers, which will provide a much more reliable approach. The main objective of this study was to compare the prevalence of consumption during pregnancy as assessed by a questionnaire and a hair biomarker. (2) Method: A cross-sectional study with a random sample of 425 pregnant women treated in public hospital consultations in Seville (Spain) and in the 20th week of their pregnancy, orally interviewed using an elaborated ad hoc questionnaire that evaluated variables of sociodemographic, obstetric, and alcohol consumption. Furthermore, the ethyl glucuronide metabolite (EtG) was tested on a hair sample in 252 pregnant women who agreed to facilitate it. Once the data obtained through the questionnaire and hair test were analyzed, the level of metabolites and self-reported alcohol consumption were compared. (3) Results: The prevalence of self-reported alcohol consumption (questionnaire) was 20.7%, and the real consumption (metabolite analysis) was 20.2%. In 16.8% of pregnant women who declared not consuming alcohol during their pregnancy, noticeable consumption was detected according to the metabolite test. No relevant level of variability in estimated alcohol consumption was detected in the biomarker with respect to the sociodemographic and obstetric variables studied. (4) Conclusions: The prevalence of alcohol consumption during pregnancy obtained through both questionnaires and metabolite analyses was similar and high. There is no association between consumption and sociodemographic factors in this sample. The determination of consumption through biomarkers allows for a more accurate approximation of the prevalence of consumption than estimated through questionnaires. Larger sample-sized studies are needed to determine consumption patterns and thus guide the adoption of more precise policies fostering abstinence from alcohol consumption since the preconception period.

## 1. Introduction

The negative effect of alcohol consumption during pregnancy is concerning and has been gradually described in the scientific literature since the second half of the past century, with increasing evidence until today [1,2]. The term fetal alcohol spectrum disorder (FASD) is nowadays used to describe a group of cognitive disorders, sensory processing disorders, and dysmorphologies resulting from prenatal exposure to alcohol. Alcohol consumption during pregnancy is also considered the main, non-genetic, and preventable cause of intellectual disability in developed countries [3].

Despite the fact that the deleterious effect of alcohol consumption during pregnancy is dose-dependent, there is no evidence for a minimum secure dose of alcohol intake during pregnancy, and therefore abstinence is the most effective preventive strategy [4]. However, the global prevalence of alcohol consumption during pregnancy has recently been established at 9.8% [5], although the prevalence of alcohol consumption during pregnancy in both Europe and the United States of America is significantly higher [6]. There is also a low perception of risk among expectant mothers regarding the dangers of alcohol consumption during pregnancy, in particular among the youngest pregnant women with respect to beer, as well as those with a low educational level with respect to both beer and distilled spirits [7].

Prenatal alcohol exposure is a major concern and particularly prevalent in Europe and North America. Despite the serious health risks associated with alcohol consumption during pregnancy, its prevalence has been understudied to date, which indicates the need for more accurate and reliable results [8,9]. This is because most population studies gathering consumption prevalence data are based on self-reported data provided by pregnant women in response to a questionnaire. This may lead to an underestimate of consumption prevalence as a result of potential recall and social desirability biases, as well as underreporting of risky behaviors for fear of social stigmatization or the possibility of losing the child to social services [10,11,12].

Therefore, to improve our understanding of the problem, reliable data are necessary on the prevalence of alcohol consumption during pregnancy for research purposes to measure the extent of the problem. There is an incipient trend to obtain these data from the analysis of biological samples from the pregnant woman (hair) and the newborn (meconium) obtained during pregnancy or after birth, respectively [13]. The use of biomarkers is becoming increasingly useful, as this method provides accurate information with respect to time and consumption amounts, and it can be replicated, allowing us to detect not only high levels of consumption but low levels or the total absence of the biomarker, indicating abstinence from the pregnant woman [13,14]. Several biomarkers have been used in recent years to detect alcohol consumption during pregnancy in biological matrices—such as blood, urine, meconium, and hair—but to gain access to knowledge on regular consumption during pregnancy, it is preferable to determine the EtG (ethanol minor metabolite detoxified in the maternal liver) present in the hair of the pregnant woman [15]. The hair of pregnant women as a biomatrix has the advantage of determining alcohol consumption during pregnancy by knowing the amount and chronology. Detection in meconium only covers the last two trimesters of pregnancy. Unfortunately, the analytical methodology is not readily available in all services but should be considered in those cases of suspected consumption, although patients deny it in the questionnaire [16].

Thus, the gathering of reliable data (a precise amount of alcohol consumption in pregnancy) to measure the extent of the problem (the prevalence of alcohol consumption in pregnancy) allows a more precise estimation of alcohol consumption during pregnancy. A realistic balanced vision of the prevalence of alcohol consumption will help to assess the problem dimension, establish preferred target groups in the preventive intervention, and plan the allocation of health and social resources that address both the problem of alcohol consumption during pregnancy as well as the health, educational, and social response to FASD cases and their families. Furthermore, it would also help to increase social awareness of the seriousness of the phenomenon of alcohol consumption during pregnancy. The usefulness of an accurate estimate of the prevalence of alcohol consumption in pregnancy will allow for the identification of pregnant women who consume alcohol, and it could facilitate the adoption of appropriate care strategies for high-risk pregnancies. Previous studies have identified previous consumption as one of the most relevant predictor factors [17]. This is why preventive strategies should not be exclusively limited to pregnant women but cover all women of reproductive age.

For all these reasons, a study has been conducted to establish the prevalence of prenatal alcohol consumption, the degree of agreement between the information on alcohol consumption obtained through the questionnaire (self-reported consumption), and the result of the analysis of a biomarker present in the samples of maternal hair (real consumption). Furthermore, this study aims to identify the sociodemographic and obstetric characteristics of pregnant women that indicate real consumption and to recognize those pregnant women who may need special support from health services, which could serve as a basis for defining more active, specific, and effective preventive social and health strategies.

## 2. Materials and Methods

### 2.1. Study Design

A cross-sectional study was carried out with a random sample of pregnant women cared for in outpatient consultations at a public hospital in Seville (Spain). Selection was carried out when they attended their 20-week ultrasound morphology scan during a period of 5 months in 2016. All pregnant women who accepted to participate in the study (425) were interviewed orally by a health professional using a questionnaire. Furthermore, they were asked to provide a hair sample, to which 261 of them agreed. 

### 2.2. Data Collection and Participants

Regarding self-reported alcohol consumption and sociodemographic and obstetric factors, data collection was performed through a hetero-administered questionnaire conducted by health professionals trained in this specific task. The anonymity and confidentiality of the participants were guaranteed during the entire process. The questionnaire was designed ad hoc by the research team with pilot testing, which allowed for the assessment of its internal validity.

**Figure 1:**
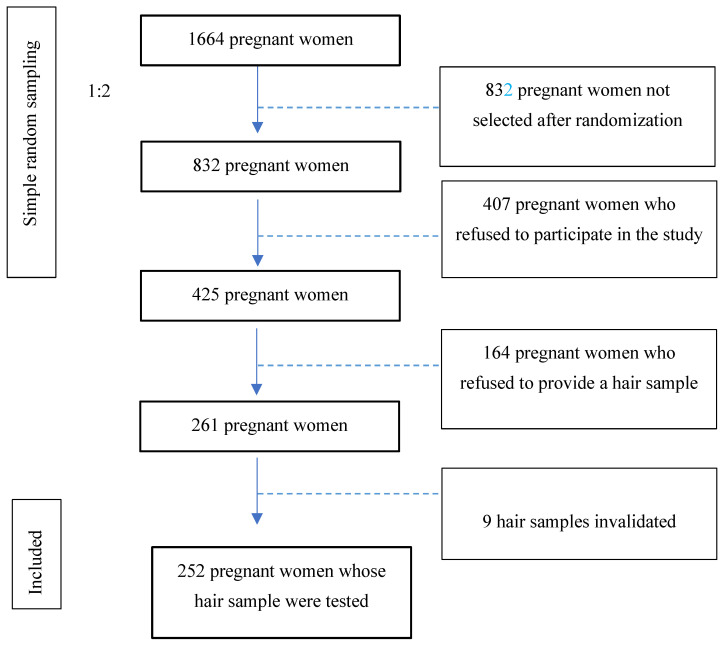
Study patient flow.

With respect to participants, the desired sample size was 400. In the overall study, the sample size was calculated using the G * power program, considering a heterogeneity equal to 50% and an α-level of 0.05. The inclusion criteria met by pregnant women who agreed to participate in the study were to be between 19 and 22 weeks of pregnancy, 16 years of age or older, who could understand and speak fluent Spanish, and who accepted to sign the informed consent form. Figure 1 illustrates the flow of patients. 

### 2.3. Maternal Hair Sample Collection

After the anatomy scan, one of every two pregnant women following a simple random sampling of 1:2 to avoid the selection bias (number of patients at the start: 1664 patients ≥ 832 pregnant women after 1:2 randomization) was invited to participate in the study by answering an anonymous questionnaire and, if they consented, by providing a hair strand. This hair sample had to meet the following requirements: having a length of at least 6 cm, so it could chronologically comprise the entire period studied (from the start of pregnancy). The weight amount necessary to complete the analysis was over 17 grams. The hair sample was collected by cutting the strand of hair as closely as possible to the scalp in the occipital area. Pregnant women who refused to provide a hair sample (38.6%) were not asked for the reason for their refusal. 

### 2.4. Ethical Aspects

The study protocol was approved by the hospital ethics committee (Código: ICG15/internal code: 025N-15). All pregnant women who participated in the study were informed of the objectives through interviewers and, after reading informed consent and determining the resolution of the doubts, were asked to provide their written informed consent. The participants received a copy of the same document. Confidentiality and anonymity preservation were always guaranteed by means of a code assigned to each participant in the study. This research complies with the universal guidelines of the Declaration of Helsinki of 1975 and subsequent amendments.

### 2.5. Questionnaire 

As discussed above, the questionnaire was developed ad hoc on the basis of the previous literature on factors related to alcohol consumption during pregnancy [11,18]. A multidisciplinary team of health professionals (a primary care physician, a gynecologist, a midwife, and a neonatologist) and professionals from the fields of psychology and sociology were the authors of the Del questionnaire. Although the questions had a closed format with multiple choice, there was the possibility of writing the answers spontaneously provided by the participants, pregnant women, in the section labeled ‘other’. Subsequently, the research team classified these responses. 

### 2.6. Variables

The questionnaire included the following groups of variables:

- Sociodemographic variables: age (categorized in <25 years; 26–30 years; 31–35 years, more than 35 years old), education level (none, primary, secondary, university), employment situation (stable, vulnerable—defined as the sum of employment status groups of own-account workers and contributing family workers—self-definition as “housewife”, or other) if the pregnant woman has a partner or not, her country of origin (Spain or another country), size and weight (used to calculate body mass index according to the World Health Organization, classified as thinness (below 18.5), normal weight (18.6–24.9), pre-obesity (25–29.9), class 1 obesity (30–34.9), and class 2–3 obesity regrouped (more than 35)).
- Obstetric variable: number of pregnancies, including the current one, classified as 1 or more than one.
- Alcohol Consumption-Related Variables

- Average number of grams of pure alcohol consumed per day during pregnancy, according to self-reported alcohol consumption (including days without consumption). From a selection of items from the AUDIT screening tool [18], the average amount of alcohol grams consumed per day was estimated in each pregnant woman according to the frequency of consumption of each type of beverage, their average level of alcohol, and the usual volume of each type of glass used to drink them in the city of Seville. Subsequently, this variable (grammes of pure alcohol per day consumed during pregnancy) was classified into three values: non-consumption, lower than or equal to 1.96 g/day, and more than 1.96 g/day, being 1.96 g, the average amount consumed per day by pregnant women who declared to drink alcohol during their pregnancy [19].
- Alcohol consumption was determined from the analysis to detect the EtG metabolite, evaluating the concentration of pg/mg in hair. This variable was categorized into 3 groups: Negative: no EtG detected; Abstinence (<5 pg/mg); and Consumption (>5 pg/mg).

### 2.7. Hair Analysis for the Detection of Alcohol Consumption (EtG)

EtG was measured in each of the hair segments using a previously published method and re-validated in-house with some analytical improvements [20]. Briefly, aliquots of 25 mg of finely cut hair were extracted after 1 h of incubation at 100 °C in 0.5 mL of an acidic buffered M3 reagent containing 1.5 ng/mL of EtG-d5. Then, the extracted hair was dried under nitrogen flow and re-suspended in 0.1 mL of mobile phase A. After vortex mixing and ultracentrifugation at 10.000 g for ten min, 10 μL of the clear supernatant were injected into UHPLC-MS/MS. Chromatography was carried out using a Luna Omega Polar C18 (100 × 2.1 mm, 1.6 μm) using a linear gradient elution with two solvents: 0.1% formic acid in water (mobile phase A) and methanol (solvent B). Solvent B was maintained at 2.0% for the first 0.5 min. It was increased to 95.0% from 0.5 to 2.0 min and held to 95.0% from 2.0 to 2.5 min. Then it was decreased back to 2.0% from 2.5 to 2–6 min and held to 2.0% from 2.6 to 5 min to re-equilibration. The flow rate was kept constant at 0.3 mL/min during the analysis. EtG and the internal standard (EtG-d5) were detected in negative electrospray ionization mode with the triple quadrupole mass spectrometer operated in multiple reaction monitoring (MRM). Mass spectrometry conditions were the following: capillary voltage of 2.5 kV, desolvation temperature of 650 °C, source temperature of 150 °C, cone gas flow rate of 40 L/h, desolvation gas flow rate of 900 L/h, and collision gas flow rate of 0.07 mL/min. The cone energy voltage was 20 V, and the collision energy voltages were 15 and 18 eV for both EtG and EtG-d5. MRM transitions were: *m*/*z* 221.1→75.0, 221.1→85.0 for EtG, and *m*/*z* 226.0→75.0, 226.0→85.0 for EtG-d5. Underline transitions were selected for quantification. The limit of quantification was 5 pg/mg.

Both EtG and the internal standard, ethyl glucuronide-d5 (EtG-d5), used in this study were purchased from Cerilliant (Austin, TX, USA). M3 (acidic aqueous buffer) reagent was provided by Comedical S.a.s. (Mattarello, Trento, Italy). HPLC-MS-grade solvents (methanol, acetonitrile, and water) were purchased from Carlo Erba (Milan, Italy). All other chemicals used for experiments were analytical reagents or HPLC-grade from commercial resources.

According to the latest international consensus on the use of alcohol markers in hair for the assessment of abstinence and chronic alcohol consumption [21,22], a concentration lower than or equal to 5 pg/mg EtG in the proximal head hair segment (3–6 cm) does not contradict self-reported abstinence. Conversely, a concentration greater than 5 pg/mg EtG in the proximal head hair segment strongly suggests repeated alcohol consumption, commonly defined as “social drinking”. A concentration greater than or equal to 30 pg/mg EtG in the proximal head hair segment with a length of 3 cm up to 6 cm strongly suggests chronic excessive alcohol consumption.

### 2.8. Data Analysis

First, a univariate analysis of all the variables included in the study was conducted to evaluate the percentage distribution of the different response categories. Second, self-declared alcohol consumption during pregnancy was compared with the hair analysis of the EtG biomarker. Third, bivariate analyses of the relationships between the categorical variables of alcohol consumption and the sociodemographic or obstetric variables were performed using a chi-square test χ^2^ and its nonparametric variants with the corresponding contingency tables. Cramer’s V was calculated for effect sizes. The statistical software package SPSS version 21.0 was used to conduct these analyses.

## 3. Results

### 3.1. Descriptive Analysis of the Sample

As introduced in a previous section, the total number of participants in the study was 425 pregnant women, of whom 261 (61.4%) provided hair samples and only 252 (59.3%) were analyzable. Moreover, 38.6% of the pregnant women who participated in the study did not provide a hair sample, spontaneously and mainly for aesthetic reasons. Of those who accepted to facilitate a hair strand, 51 cases showed positive EtG and therefore consumed alcohol during pregnancy. Most pregnant women were born in Spain (92.2%), and the average age was 31.9 (SD = 5.3). The descriptive information of the sample according to the obstetric and demographic variables can be found in Table 1.

### 3.2. Alcohol Consumption in Pregnant Women, According to Metabolite Analysis

**Figure 2:**
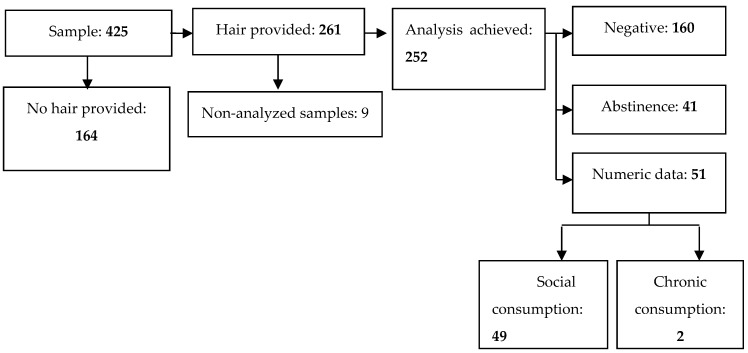
Case flow according to metabolite analysis.

Of a total of 425 pregnant women, 252 provided a hair strand to be tested (nine of them did not reach the amount of grams needed to be processed). In 160 of these hair samples, the results were negative, and 41 showed minimum negligible levels, which were classified as abstinence. Furthermore, 51 pregnant women self-reported their alcohol consumption during pregnancy, obtaining a prevalence of real alcohol consumption of 20.2%. The hair analysis flow chart is illustrated in Figure 2, and the consumption is described in Table 2.

Forty-one pregnant women with a minimum EtG level were detected, and the results were classified as abstinence. On the other hand, with respect to the two pregnant women showing chronic consumption, the metabolite analysis determined a concentration of 68.7 pg/mg and 73.4 pg/mg, respectively.

### 3.3. Comparison between Self-Reported Consumption and the One Detected in the Metabolite

Table 3 shows the results of the cross-tables using the metabolite method as the gold standard to validate the self-report measure. The prevalence of self-reported alcohol consumption obtained through the questionnaire was 20.7% (15.4% consuming up to 1.96 g of pure alcohol per day on a daily average, +5.3% consuming more than 1.96 g of pure alcohol per day on a daily average, and 1.96 grammes being the estimate of daily average alcohol intake consumed by pregnant women users according to the calculations based on their testimonies). A contrast was made between the self-reported consumption indicated in the questionnaire and the metabolite analysis (EtG) performed.

**Figure 3:**
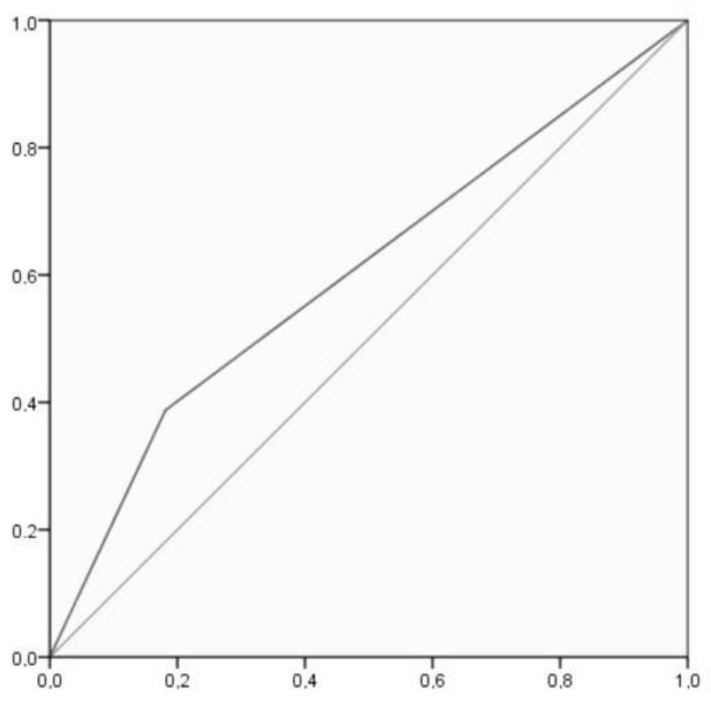
ROC curve, where the X axis represents specificity and the Y axis represents sensitivity.

Results indicated that the vast majority of the 179 pregnant women who reported not consuming alcohol showed a negative result in the metabolite analysis (122 cases) or such a lower level that may be considered an indicator of abstinence (27 cases), as illustrated in the left section of Figure 3. A minority of 30 participants who declared not drinking alcohol during their pregnancy showed indicators of alcohol consumption in their results. 

In turn, among pregnant women who declared alcohol intake during pregnancy (52 in total), the cases are divided between those presenting a level of metabolite high enough to indicate consumption, those in which the metabolite was detected but at low levels, and those whose EtG analysis resulted in negative results and therefore indicated non-consumption. A total of 23 pregnant women (14 + 9) represents the latter scenario.

In general, there is a significant association between the level of alcohol consumption according to the analysis of metabolites and the average daily intake of pure alcohol in grams estimated from the questionnaire, both considered categorical variables, with significant results (χ^2^ (4) = 15.10, *p* = 0.004, Cramer’s V = 0.18).

Sensitivity (i.e., the self-report measure’s ability to designate an individual with consumption in the EtG measure) and specificity (i.e., the self-report measure’s ability to designate an individual who does not have consumption in the EtG measure as negative) analyses were conducted. For these analyses, a 2 × 2 cross table (Table 4) was created by merging in EtG the categories negative and abstinence and in self-report the categories under and over 1.96 g/day. The sensitivity test showed a 38.78% [25.20–53.76]: true positive/(true positive + false negative) = 19/19 + 30. The specificity test indicated an 81.87% [75.49–87.18]: true negative/(true negative + false positive) = 149/149 + 33. The positive likelihood ratio is 2.14 [1.34–3.42], and the negative likelihood ratio is 0.75 [0.59–0.94]. Accuracy is 72.73% [66.50–78.36]. ROC analysis showed an AUC = 0.603, CI [0.509–0.697], *p* = 0.027, which indicated poor discrimination of the self-report measure. Figure 3 shows that there was a 60.35% chance that the self-report measure correctly distinguished alcohol consumption based on the EtG result.

### 3.4. Sociodemographic and Obstetric Variability of Alcohol Consumption Evaluated through Metabolite Analysis

No differences in relation to sociodemographic and obstetric characteristics were observed between the groups of pregnant women whose hair analysis was positive, those cases in which it was negative, or those with minimum EtG levels detected, as illustrated in Table 5 with the robust contrast statistic. However, some significant results were found when comparing groups with Z tests. Thus, more consumption was observed in housewives (Z = 2.9), less negative consumption was detected in women with primary studies (Z = −2.3), and less abstinence was found in women with overweight (Z = −2.1).

## 4. Discussion

The aim of this study is to establish the degree of agreement between the information on alcohol consumption obtained via questionnaire (self-reported consumption) and the result of the analysis of a biomarker present in the samples of maternal hair (real consumption). Similarly, the study aims to determine whether sociodemographic and obstetric factors present relevant variability according to metabolite detection or not. To do so, a random sample was used, representative of pregnant women attending antenatal screening care in an outpatient visit at a hospital in the south of Spain (Seville) during their 20th week of pregnancy.

The real prevalence of alcohol consumption obtained in this sample of pregnant women through the analysis of EtG (the direct metabolite of ethanol) in a maternal hair sample was 20.2%, while the one obtained through the questionnaire was 20.7%. Both prevalences (estimated from the metabolite and self-reported) show similar percentages, indicating that in this study there is strong agreement in the estimation of alcohol consumption during pregnancy through both methods used. Studies that analyze real alcohol consumption in pregnant women through biomarkers (hair or meconium) compared to self-reported consumption show not so coincident results, as seen in the review by Chiandetti and collaborators, which included 13 studies from different countries (Spain, Italy, Denmark, USA, Canada, Sweden, and Uruguay) that analyzed biomarkers in hair, meconium, urine, and serum, findings that determined that the estimated prevalence of alcohol consumption during pregnancy obtained through a questionnaire ranged from 0 to 37%, while the prevalence of real consumption analyzed through a metabolite ranged from 16 to 44% [16]. Therefore, the use of biomarkers to assess the consumption of toxic substances during pregnancy in general and alcohol in particular can be of great help, as self-reported consumption tends to be underestimated because of potential recall and social desirability biases, fear of stigmatization, and under-reporting of risky behaviors.

In the present study, there was a group of pregnant women (30 pregnant women) who declared that they had not consumed alcohol during pregnancy, although the metabolite analysis detected consumption. This fact may be because the declaration of consumption by pregnant women is not always correlated with reality and becomes an unreliable source for reasons such as shame, social desirability, and recall bias [23]. On the other hand, among the pregnant women who declared their alcohol consumption during pregnancy and provided a hair sample (23 of them), the metabolite analysis indicated that there was none. This circumstance could be related to the limitations of the EtG biomarker EtG measurement technique, as some pathologies, such as diabetes and liver and kidney diseases, may alter the cut-off point [23]. Another circumstance that could be related to the drop in EtG levels until undetectable may be hair bleaching or permanents [24]. Regarding the two patients in whom the metabolite analysis showed chronic consumption (73.4 pg/mg/68.7 pg/mg), their ages were allocated beyond 2DS from the mean age (mean: 31.9 ± 5.3 DS; patients’ ages: 38 and 18 years, respectively). These circumstances may be determined by the fact that younger women may have a low-risk perception of alcohol consumption during pregnancy [7], and older and multiparous women may underestimate the harmful effects of alcohol consumption during pregnancy [25].

In this study, no association was found between alcohol consumption during pregnancy evaluated using the biomarker and the included sociodemographic or obstetric variables. While studies determining self-reported consumption allowed establishing consumption patterns based on the sociodemographic and obstetric factors of pregnant women [26,27,28], no studies have been found in which real alcohol consumption measured through biomarkers allowed determining the sociodemographic or obstetric profile of pregnant women who consume alcohol. This could be because detection of alcohol consumption during pregnancy from biological samples is difficult and prevents obtaining sample sizes that allow one to determine differences in profiles among pregnant women. However, there are studies that establish consumption patterns when different psychoactive substances are associated with biomarkers derived from cannabis, alcohol, and cocaine, as the consumption of several toxic substances during pregnancy may be related, and therefore it could be possible to determine consumption patterns among pregnant women [29].

Although alcohol consumption during pregnancy could have been considerably reduced by the abstinence policies developed, there is still a long way to go, as not all actions launched by health institutions to reduce consumption reach pregnant women at higher risk. Therefore, it is necessary to evaluate the social context of pregnant women in relation to consumption habits before pregnancy [30,31], as it is considered one of the most relevant predictors of alcohol consumption during pregnancy. This is why preventive strategies should include women of reproductive age or in the periconception period so that prenatal care could modify harmful risk factors [32].

The social and health care approach of those responsible for the health and toxic substances use policies addressed to women during pregnancy is crucial to designing and implementing preventive health strategies. First, it is important to provide health professionals with the appropriate knowledge to screen and detect potential consumers of pregnant women and provide the appropriate health advice adapted to the knowledge and beliefs of pregnant women. A recent review published by Dahl et al. indicates that health professionals have neither the appropriate knowledge nor the tools necessary for screening [33]. Furthermore, pregnant women may receive inconsistent health advice on alcohol consumption during pregnancy, even those indicating that social alcohol consumption is acceptable [33]. This is in line with other articles that indicate that the training of healthcare professionals is crucial to providing quality and personalized health advice to pregnant women [34]. However, the preventive strategy of the alcohol consumption screening strategy, together with the real determination through biomarkers during pregnancy so that it could be included as a record history in the newborn, can lead to sensitive circumstances in prenatal care since it could reduce the autonomy and trust of the pregnant woman with health professionals [35].

Regarding the strengths of the study, it can be highlighted that the sample used was homogeneous (same gestational age) and a random sampling of pregnant women who attended an outpatient clinic was carried out. The questionnaire was administered in person by health professionals with specific training for this purpose. In addition to the questionnaire, EtG was used as a biomarker in the hair sample to determine the prevalence of alcohol consumption. The limitations of the study include the fact that it is observational work, so it is not possible to establish inferences about causal relationships between variables. The questionnaire and hair sampling were performed in the first half of the pregnancy (week 20), so it was not possible to assess what might appear after that week. Pregnant women who did not have a fluent level of Spanish to understand the questions on the questionnaire and answer them could not be included in the study since no translation service was available. It is not discarded that there might have been selection bias, as the participation rate was 51.2%. Furthermore, as discussed in Section 2.3, the reason for the refusal of pregnant women to provide the hair sample was not investigated. It can be inferred that, in most cases, the refusal to provide a hair sample was due to aesthetic reasons or to reluctance to provide a biological sample, and perhaps to feelings of guilt in those pregnant women who had consumed alcohol and did not know what to give their hair sample for this reason. In any case, if the reason for the refusal had been required, probably a larger number of pregnant women would not have provided the hair sample.

## 5. Conclusions

This study on the prevalence of alcohol consumption during pregnancy obtained through a questionnaire and biomarker analysis shows that this prevalence is similar and significantly high. Alcohol consumption during pregnancy determined by biomarkers allows for a more real and precise approximation to consumption prevalence than questionnaires (avoiding underestimation and using reliable data). No relevant differences were detected in relation to sociodemographic and obstetric characteristics between pregnant women among consumers and non-consumers. More studies with a larger sample size are required to establish a profile of the pattern of alcohol consumption in women during pregnancy. However, alcohol consumption before pregnancy is a strong predictor of alcohol consumption during pregnancy. By jointly assessing all the predictive factors of alcohol consumption during pregnancy, institutions could establish tailored abstinence policies with the aim of preventing alcohol consumption and, consequently, FASD.

## Figures and Tables

**Table 1:** Description of the sample according to sociodemographic characteristics and obstetric history.

Variables	Categories	Percentage	n
Age	Up to 25 years old	12.5	53
	26–30 years old	23.3	99
	31–35 years old	37.9	161
	>35 years old	26.4	112
Educational level	None	2.4	10
	Primary	37.9	161
	Secondary	22.1	94
	University	37.6	160
Employment situation	Stable	39.4	167
	Vulnerable	45.4	193
	Housewife	5.9	25
	Other	9.2	39
Partner	Yes	98.1	417
	No	1.9	8
Country of origin	Spain	92.2	390
	Other	7.8	33
BMI	Thinness (<18.5)	0.2	1
	Normal weight (18.6–24.9)	52.3	218
	Overweight (25–29.9)	28.8	120
	Class 1 Obesity (30–34.9)	13.2	55
	Class 2–3 Obesity (>35)	5.5	23
Number of pregnancies (including the current one)	One	40.5	171
	More than one	59.5	251

**Table 2:** Determination of alcohol real consumption obtained through metabolite analysis (EtG).

No Consumption	N (%)
Negative	160 (63.5%)
Minimum levels of EtG, comparable to abstinence	41 (16.3%)
**Consumption**	
Social drinker	49 (19.4%)
Chronic consumption	2 (0.8%)
**Prevalence of consumption**	**51 (20.2%)**

**Table 3:** Levels of alcohol consumption based on self-reported alcohol consumption (average grams of pure alcohol consumed during pregnancy) in contrast with metabolite analysis.

			EtG Categorized Consumption			Total
			Negative	Abstinence	Consumption	
**Self-reported consumption**	**0 g/day**	**N**	**122**	**27**	**30**	**179**
		**%**	**68.2**	**15.1**	**16.8**	**100**
		**Adjusted residuals**	**3.1**	**−0.7**	**−3.1**	
	**<1.96 g/day**	**N**	**14**	**7**	**16**	**37**
		**%**	**37.8**	**18.9**	**43.2**	**100**
		**Adjusted residuals**	**−3.4**	**0.5**	**3.6**	
	**>1.96 g/day**	**N**	**9**	**3**	**3**	**15**
		**%**	**60**	**20**	**20**	**100**
		**Adjusted residuals**				
**Total**		**N**	**145**	**37**	**49**	**231**
		**%**	**62.8**	**16**	**21.2**	**100**

**Table 4:** A 2 × 2 cross table of frequencies of alcohol consumption according to metabolite analysis and self-reported measures.

	EtG No Consumption n	EtG Consumption n
Self-reported No Consumption	149	30
Self-reported Consumption	33	19

**Table 5:** Sociodemographic and obstetric characteristics of estimated alcohol consumption based on the analysis of the metabolite (EtG).

		Negative n (%)	Abstinence n (%)	Consumption n (%)	χ^2^	*p*	Cramér’s V
Age					1.37	0.968	0.052
	Less than 25	21 (63.6)	7 (21.2)	5 (15.2)			
	26–30	41 (63.1)	11 (16.9)	13 (20)			
	31–35	60 (62.5)	(15) 15.6	(21) 21.9			
	>35	38 (65.5)	8 (13.8)	12 (20.7)			
Country of origin					4.58	0.101	0.135
	Spain	148 (64.1)	40 (17.3)	43 (18.6)			
	Other	11 (57.9)	1 (5.3)	7 (36.8)			
Employment situation					10.31	0.112	0.143
	Stable	64 (62.7)	18 (17.6)	20 (19.6)			
	Vulnerable	75 (68.2)	18 (16.4)	17 (15.5)			
	Housewife	8 (40)	3 (15)	9 (45)			
	Other	13 (65)	2 (10)	5 (25)			
Partner					1.53	0.465	0.078
	Yes	156 (63.4)	41 (16.7)	49 (19.9)			
	No	4 (66.7)	0	2 (33.3)			
Educational level					6.56	0.363	0.114
	None	5 (83.3)	0	1 (16.7)			
	Primary	55 (55)	21 (21)	24 (24)			
	Secondary	34 (68)	8 (16)	8 (16)			
	University	66 (68.8)	12 (12.5)	18 (18.8)			
Number of pregnancies					2.81	0.246	0.106
(including this one)	One	61 (64.2)	19 (20)	15 (15.8)			
	More than one	99 (63.1)	22 (14)	36 (22.9)			
BMI					11.04	0.199	0.148
	Thinness	0	0	1 (100)			
	Normal weight	89 (64)	26 (18.7)	24 (17.3)			
	Overweight	45 (64.3)	6 (8.6)	19 (27.1)			
	Class 1 obesity	18 (62.1)	7 (24.1)	4 (13.8)			
	Class 2–4 obesity	8 (61.5)	2 (15.4)	3 (23.1)			

## Data Availability

The data are bot publicly available due to privacy restrictions, but are available from request form the corresponding authors.
